# Distorted self-perceived weight status and its associated factors among civil servants in Tamale, Ghana: a cross-sectional study

**DOI:** 10.1186/2049-3258-71-30

**Published:** 2013-11-06

**Authors:** Victor Mogre, Prosper P Mwinlenna, Jeremiah Oladele

**Affiliations:** 1 Department of Human Biology, School of Medicine and Health Sciences, University for Development Studies, Tamale, Ghana; 2 Department of Community Nutrition, School of Medicine and Health Sciences, University for Development Studies, Tamale, Ghana

**Keywords:** Weight Status, Civil Servant, Central Obesity, Major Public Health Concern, Weight Perception

## Abstract

**Background:**

Obesity has been described as an epidemic and a major public health concern globally. Distorted self-perceived weight status can negatively impact an individual’s decision to lose weight as well as adoption of healthful weight management attitudes. This study described self-perceived weight status among adults working in civil service departments in Tamale, Ghana, and compared it to their classification based on *WHR*. It also examined associations of distorted self-perceived weight status with weight loss attitudes, socio-demographic variables and knowledge levels on the health effects of overweight and obesity.

**Methods:**

This cross-sectional study was undertaken from January 2011 to July 2011 among a sample of 186 civil servants living in Tamale. Out of the sample, 121 were men and 65 were women. Participants’ self-perceived weight status, socio-demographic and weight loss attitudes were assessed by means of a 10-item questionnaire. Participants’ waist and hip circumferences were measured with appropriate tools and computed into *waist hip ratio (WHR*) and classified based on WHO classifications.

**Results:**

More than 80% of the participants were aged below 40 years. Generally, 56.5% (n = 105) participants had *normal weight* and 31.2% (n = 58) were *centrally obese*. The proportion of participants being *centrally obese* was higher in women compared to men (p < 0.0001). Forty four percent of the studied population had a distorted self-perceived weight status. Less than10% of participants self-perceived themselves as overweight/obese, in which over 47% were, in fact, *overweight/obese* as measured by WHR. Factors associated with distorted self-perceived weight status were being *overweight/obese* (Crude OR = 97.3; (35.8-264.6; p < 0.0001), aged < 40 years (Crude OR = 2.8; 95% CI = 1.3-6.5; p = 0.0102) and having inadequate knowledge on the health effects of overweight/obesity (Crude OR = 3.7; CI = 1.3-11.0; p = 0.0114). Weight loss attitudes and methods used to lose weight were not significantly associated to self-perceived weight status and *WHR* measured weight status.

**Conclusions:**

Self-perceived and *WHR* measured weight status of participants did not conform. Distorted self-perceived weight status was not associated to weight loss attitudes but to being *overweight/obese*, being younger (<40 years) and having inadequate knowledge on the health effects of overweight/obesity. Educating people on accurate weight perception and the health effects of overweight/obesity should be considered in designing public health strategies to curb the rising prevalence of overweight/obesity and other non-communicable diseases in Ghana.

## Background

Obesity has been described as an epidemic, an international problem and a major public health concern globally [1, 2]. In the UK, the prevalence of obesity was reported to be 23% among men and 24% among women in 2006 [3]. Over the last decade, recent studies indicate that the prevalence of obesity in the US has increased from 12.0% to 19.8% [4, 5] and half of its adult population is now overweight or obese [6]. However, it is no longer a disease of industrialized countries but exist in developing countries as well [7]. An obesity prevalence of 10% has been reported in West Africa [8] and specifically 18% in the Republic of Benin [8]. In Ghana, a study conducted by Owiredu et al., [9] in Kumasi, Ghana, reported the prevalence of central obesity to be 27.9% among sedentary workers. Several studies have revealed that the prevalence of obesity in Ghana is increasing especially among women [10–15].

Though several factors have been attributed to the current situation, including urbanization, globabalization, nutritional transition and sedentary lifestyles [7, 13, 15–18], this study intended to examine the potential contribution of alterations of self-perceived weight status and objective weight. In the present study, a distorted weight perception occurs when one self-perceives his/her weight to be found in a different category (normal weight, overweight, obese) than would have been if determined by an objective method of measurement [19]. Self-perception of one’s weight status may differ from both public health and normative cultural standards. In African countries including Ghana, fatness has been associated with wealth, prosperity, affluence and happiness [20, 21]. Mvo et al., [21] reported that among the black South African population, obesity has no negative social connotations.

Distorted self-perceived weight status has been extensively studied elsewhere, though has not been adequately investigated in Ghana [19, 22–25]. Several studies have demonstrated a strong association between self-perceived weight status and weight control behaviour, independent of objective weight status [26–30].

This study investigated the weight perception of weight among adults working in civil service departments in Tamale, Ghana and compared to their weight classifications based on *WHR*. Furthermore, the study sought to examine the associations between distorted self-perceived weight status and weight loss attitudes (i.e. having tried to lose weight and methods used) and some demographic variables (sex and age) and knowledge on the health effects of overweight and obesity. Exploring the relationship between self-perceived weight status and objectively measured weight status as well as weight control attitudes will be potentially useful in the holistic approach to help tackle the rising prevalence of obesity and other non-communicable diseases in Ghana.

## Methods

### Participants

This cross-sectional study was conducted between January and July, 2011 among adult civil servants living and working in civil service departments in Tamale, Ghana. Tamale is the capital city of the Northern region of Ghana. It lies between latitude 9°22′N and longitude 0°50′W covering an area of about 922 km^2^. It is about 600 km north of Accra, the capital city of Ghana. The Civil Service forms part of the Public Service of Ghana [31]. There are 13 civil service departments in Tamale with a workforce of about 2500. Three departments were randomly selected from a list of the 13 civil service departments. Participants were selected using a proportionate random sampling technique that included more participants from larger departments, with the help of random number statistical tables. From each selected department, a proportionately determined number of participants were selected from the list of potential participants in that department. Selected personnel who declined to participate or were unavailable to participate were immediately replaced with others from the department.

The inclusion of participants was voluntary and informed consent was obtained from each participant. Participants were also assured of confidentiality of any data taken. A total of 186 out of the 265 (70.2% participation rate) civil servants working in the three sampled civil service departments participated in the study.

### Ethical approval

The study was approved by the Ethics Committee of the University for Development Studies, School of Medicine and Health Sciences, Ghana.

### Anthropometric variables

Anthropometric measurements included waist circumference, measured midway between the inferior angle of the ribs and the suprailiac crest. Hip circumference, measured as the maximal circumference over the buttocks in centimetres. Both measurements were measured to the nearest 1 cm using a non-stretchable fibre-glass measuring tape (Butterfly, China). During both measurements, subjects were made to stand upright, with arms relaxed at the side, feet evenly spread apart and body weight evenly distributed in accordance with the WHO expert consultation report on waist circumference and waist-hip ratio (WHO, 2004). All anthropometric measurements were taken in a private room allocated for that purpose in each of the sampled departments. *Waist to Hip Ratio (WHR)* was calculated by dividing the waist circumference (cm) by the hip circumference (cm). Men with WHR < 0.90, 0.90–0.99 and ≥ 1.0 were classified as *normal, overweight or obese* respectively, while women were classified in the same categories on the basis of WHR of < 0.80, 0.80-0.84 and ≥ 0.85 [32].

### Questionnaire

A 10-item self-administered questionnaire was developed to collect information on self-perceived weight status of participants and participants’ methods of losing weight and awareness of the health effects of obesity. Weight perception was assessed using the question: “Do you think your weight is: about the right weight, overweight or obese”. Socio-demographic data such as gender, age and educational status were also asked. The questionnaire underwent pilot testing for clarity and comprehensibility with a group of 10 civil servants. Based on the outcome of the pretesting, a few minor changes were made. The questionnaire also had questions on method of weight control. Self-perceived weight status of participants was then compared with their measured WHR.

### Statistical analysis

Self-perceived weight status was compared to the measured *WHR* by cross-tabulation and Kappa statistics of agreement. Univariate analysis using simple logistic regression was used to assess influence of different variables on distorted weight perception. The results were reported as crude odds ratio (OR) at 95% confidence interval (CI). The results were expressed as proportion and compared using Fischer’s exact test or χ^2^ for trend analysis as appropriate. A level of p < 0.05 was considered as statistically significant. GraphPad Prism version 5.00 (GraphPad software, San DiegoCalifornia USA, http://www.graphpad.com/) and SPSS version 18 for windows were used for all the statistical analysis.

## Results

General characteristics of the studied participants are presented in Table 1. The study included 121 men and 65 men. By *WHR*, out of the 186 participants, 56.5% (n = 105) had *normal weight*, 12.4% (n = 23) were *overweigh*t and 31.2% (n = 58) were *centrally obese*. The prevalence of *central obesity* was significantly higher in women than in men (p = 0.0312). On the other hand, significantly more men than women had *normal weight* status (p = 0.0032).

**Table 1:** **General characteristics of the studied population (n = 186)**

Variable	Total (n = 186)	Men (n = 121)	Women (n = 65)	P value
**Age (years)**				
< 40	150 (80.6%)	94 (77.7%)	56 (86.2%)	0.1790
≥ 40	36 (19.4%)	27 (22.3%)	9 (13.8%)	
**Educational level**				
High	167 (89.8%)	108 (89.3%)	59 (90.8%)	0.8056
**Weight status measured by WHR**				
Normal weight	105 (56.5%)	78 (64.5%)	27 (41.5%)	0.0032
Overweight	23 (12.4%)	12 (10.0%)	11 (16.9%)	0.1707
Obese	58 (31.2%)	31 (25.6)	27 (41.5%)	0.0312
**Self-Perceived weight status**				
Overweight/obese	17 (9.1%)	14 (11.6%)	3 (4.6%)	0.1808
Normal weight	169 (90.9%)	107 (88.4%)	62 (95.4%)	
**Classification of Self-Perceived weight status**				
Distorted	82 (44.1%)	50 (41.3%)	32 (49.2%)	0.2622
Non-distorted	104 (55.9%)	71 (58.7%)	33 (50.8%)	
**Ever tried to lose weight**				
Yes	72 (38.7%)	41 (33.9%)	31 (47.7%)	0.0824
No	114 (61.3%)	80 (66.1%)	34 (52.3%)	
**Methods used to lose weight**				
Diet	36 (50.0%)	15 (36.6%)	21 (67.7%)	0.0167
Exercise	36 (50.0%)	26 (63.4%)	10 (32.3%)	
**Health personnel’s advised me to lose weight**				
Yes	32 (44.4%)	21 (51.2%)	11 (35.5%)	0.2337
No	40 (55.6%)	20 (48.8%)	20 (64.5%)	
**Has awareness on obesity and its health effects**				
Yes	168 (90.3%)	112 (92.6%)	56 (86.2%)	0.1949
No	18 (9.7%)	9 (7.4%)	9 (13.8%)	

*Data are presented as proportion and analyzed using Fischer’s exact test.*

While 90.9% of the studied population self-perceived themselves as normal weight, 9.1% self-perceived their weight as overweight/obese. From the studied population, more women (95.4%) than men (88.4%) self-perceived their weight as normal. Generally, 44.1% of the studied population had a distorted self-perceived weight status. There was no significant relationship between participants’ self-perceived weight status, classification of self-perceived weight status and gender. Of the studied participants, while 61.3% (n = 114) had never tried to lose weight, 38.7% had ever tried to lose weight. Exactly the same proportion of the studied participants who ever tried to lose weight used diet and exercise. While, the proportion of men using exercise to lose weight was higher than women, the proportion of women using diet to lose weight was higher than men (p = 0.0167). More men (51.2%) than women (35.5%) were advised to lose weight by health personnel, even though the differences were not statistically significant. Of the 186 studied participants, 90.3% said they had awareness on the health effects of obesity with insignificant differences between men and women.

The effect of sex, age, weight loss attitudes and *WHR* measured weight status on the risk of having a distorted self-perceived weight status is presented in Table 2. Using univariate analysis, having an age below 40 years (OR = 2.8; 95% CI = 1.3-6.5; p = 0.0102) and knowledge on the health effects of obesity (OR = 3.7; CI = 1.3-11.0; p = 0.0114) were the variables that significantly increased the risk of having a distorted self-perceived weight status. In addition, participants who were *overweight/obese*, were several folds more likely to have a distorted self-perceived weight status compared to participants measured as normal weight (OR = 97.3; 35.8-264.6; p < 0.0001) using the univariate analysis.

**Table 2:** **Univariate analysis of some factors for distortion of weight status**

Variable	n/N*	Rate of distortion	OR (CI 95%)	P value
**Sex**				
Male	50/121	41.3%	Ref	Ref
Female	32/65	49.2%	1.4 (0.8-2.5)	0.3003
**Age (years)**				
20-39	73/150	48.7%	2.8 (1.3-6.5)	0.0102
40-49	9/36	25.0%		
**Ever tried to lose weight**				
Yes	34/72	47.2%	1.2 (0.7-2.2)	0.4936
No	48/114	42.1%	Ref	Ref
**Has awareness on obesity and its health effects**				
Yes	69/168	41.1%	Ref	Ref
No	13/18	91.0%	3.7 (1.3-11.0)	0.0114
**WHR Status**				
Normal Weight	9/105	8.60%	Ref	Ref
Overweight/Obese	73/81	90.1%	97.3 (35.8-264.6)	< 0.0001

*Number of subjects with Distortion/number of subjects in each category. Ref refers to reference point.

A comparison between actual weight status as measured by *WHR* and self-perceived weight status, weight loss attitudes, awareness and age are presented in Table 3. From the over 90% of participants self-perceiving their weight as normal, 56.8% were, in fact, *normal weight*. And of the less than 10% of participants self-perceiving themselves as overweight/obese, over 47% were, in fact, *overweight/obese*. A poor strength of agreement was observed between self-perceived weight status and *WHR* (Kappa = 0.014 SE = 0.047, CI = -0.078 to 0.107). Among participants who had a distorted self-perceived weight status, whiles more than 80% of them were *overweight/obese*, only 9% had *normal weight* (p < 0.0001). Weight loss attitudes and methods used to lose weight were not significantly associated to weight status measured by *WHR*. Concerning the decision to lose weight, while, 34.4% of participants who tried to lose weight were *overweight/obese,* 65.6% had *normal weight* (p = 0.0992). By proportion, more participants aged 20-39 compared to participants aged 40-49 were *overweight/obese* (p = 0.1933).

**Table 3:** **Comparison between participants’ actual weight status (WHR) to self-perceived weight status, weight loss attitudes and age**

	Actual weight as measured by WHR			
Variable	Normal (%*)	Overweight/obesity (%*)	Total (n = 186**)	P value
**Self-perceived Weight status**				
Normal weight	96 (56.8%)	73 (43.2%)	169 (90.9%)	0.8014
Overweight/obesity	9 (52.9%)	8 (47.1%)	17 (9.1%)	
**Classification of self-perceived weight status**				
Distorted	9 (10.9%)	73 (89.0%)	82 (44.1%)	< 0.0001
Non-Distorted	96 (92.3%)	8 (7.7%)	104 (55.9%)	
**Ever tried to lose weight**				
Yes	39 (54.2%)	33 (45.8%)	72 (38.7%)	0.6507
No	66 (57.9%)	48 (42.1%)	114 (61.3%)	
**Methods used to lose weight**				
Diet	19 (52.8%)	17 (47.2%)	36 (50.0%)	1.0000
Exercise	20 (55.6%)	16 (44.4%)	36 (50.0%)	
**Health personnel’s advice to lose weight**				
Yes	21 (65.6%)	11 (34.4%)	32 (44.4%)	0.0992
No	18 (45.0%)	22 (55.0%)	40 (55.6%)	
**Has awareness on obesity and its health effects**				
Yes	95 (56.5%)	73 (43.5%)	168 (90.3%)	1.0000
No	10 (55.6%)	8 (44.4%)	18 (9.7%)	
**Age (years)**				
< 40	81 (54.0%)	69 (46.0%)	150 (80.6%)	0.1933
≥ 40	24 (66.7%)	12 (33.3%)	36 (19.4%)	

Data are presented as proportion and analyzed using Fischer’s exact test. *Row percentage, **Column percentage.

## Discussion

This cross-sectional study among Ghanaian civil servants showed a *central obesity* prevalence of 31.2%. This is consistent with the findings of Owiredu et al., [9] among Ghanaian sedentary workers and Reddy et al., [33] among an Industrial population in India. The prevalence of *central obesity* was significantly higher in women compared to men. To a large extent these findings concur with the studies of Puepet et al., [34] among a non-diabetic population in Nigeria; among an apparently healthy population in Uganda [35] and in several studies conducted in Africa [7, 36].

A high prevalence of distorted self-perceived weight status has been found in this study. Previously, higher proportions have been reported in Sub-Saharan Africa and elsewhere. A population based survey from the Seychelles reported that 72% of the studied population did not consider themselves to be overweight/obese [37]. In Morocco, 48.5% (*n* = 80) of men vs. 75.2% (*n* = 204) of women had a false perception of their body-weight status in a cross-sectional study of 436 individuals aged 20 years and above [38]. A cross-sectional survey of a sample of 493 adults in Pakistan reported that more than 70% of *obese* participants did not consider themselves as obese [39].

The high prevalence of distorted self-perceived weight status in the present study could be influenced by the predominant culture, physique of peers, media and one’s perception of body weight [40]. It has been shown that when overweight/obesity is a common feature of a cultural group, a strong negative social pressure limits involvement in weight control programmes [41]. In this part of the world, many people generally perceive overweight/obesity to be a sign of affluence. A study among University students in Nigeria showed that most subjects believed that being obese gives respect and a sign of living good [42]. As such participants misconceive themselves to be normal when, in fact they were *overweight/obese*.

In this study, a significant proportion of *overweight/obese* (in both genders) participants did not perceive themselves as overweight/obese. In fact, using univariate analysis, *overweight/obese* participants were several folds at risk of having a distorted self-perceived weight status than their normal weight counterparts. Consistent findings have been reported in a population-based survey of Seychelles adults [37], among adolescents in Nigeria [43] and among overweight/obese adults in the US [44]. Another study by Faber [45] among black women in a South African Rural village showed that 2% of Black women perceived themselves to be overweight, while in fact, 95% were overweight. Consistent with other studies, this study has demonstrated a widespread underestimation of body weight and size, especially among overweight/obese individuals [19, 25, 46].

In this study, age was found to significantly affect distorted self-perceived weight status. We found that participants aged <40 years (in both genders) had a 2.8 risk of having a distorted self-perceived weight status. Most of them underestimated their weight status to be normal when in fact they were *overweight/obese*. In contrast to our findings Bhanji et al., [39] in a study among a sample of adults in Pakistan found that participants aged > 40 years were more likely to misperceive their weight status. Furthermore, a study among male firefighters in America found that underestimating one’s weight category furthermore increased with age [47]. The inconsistencies could be attributed to social and cultural differences. In Sub-Saharan Africa being overweight/obese is a sign of good health and well-being and is generally accepted as normal weight. As a result, the younger participants can be said to be more concerned about their weight status and would like to misperceive themselves as normal according to the social and cultural norms.

Another important observation of this study was that having inadequate knowledge on the health effects of overweight and obesity was significantly associated to distorted self-perceived weight status. Participants who had inadequate knowledge had crude odds ratio of 2.7 risk of having distorted weight perception. This suggests that they did not consider overweight and obesity as risk factors for chronic diseases such as diabetes mellitus, hypertension, and coronary heart diseases. This is confirmed in a study by Gorynski et al., [48] in which people with diabetes had a poor weight perception. This is worrying in the sense that the distorted weight perception caused by having inadequate knowledge on the health effects of overweight/obesity could lead to a progressive development of obesity resulting in chronic diseases. Distorted self-perceived weight status is a potentially modifiable risk factor [44] and as such educating people on the health effects of overweight and obesity should be considered in public health interventions in the future.

Even though a high prevalence of *central obesity* has been found in this study, a large proportion of *normal weight* and *overweight/obese* participants had never attempted to lose weight. Participants appeared to be unconcerned about their weight status. In a study of black women, in South Africa few obese participants wanted to or had ever tried to lose weight [23]. Participants’ apparent poor attitude towards losing weight could be due to the fact that a large proportion of the participants in this study misperceived themselves to be normal weight, when in fact they were either overweight or obese. Self-defined body weight is very critical, as it plays a role in the weight management practices of individuals as well as their nutrition habits [49]. A study by Duncan et al., [44] reported that overweight/obese men and women who misperceived their overweight status were 60% (RR 0.40, 95% CI 0.30-0.52) and 56% (RR 0.44, 95% CI 0.32-0.59) less likely than those who accurately perceived their overweight status to have attempted weight loss during the past year. The findings of this study and several others suggest that weight perception should be considered in designing weight loss interventions [44, 50, 51].

When participants’ choice of method to lose weight was stratified by gender, more women preferred dieting as a method to lose weight while more men preferred exercise as a method to lose weight. This is consistent with the findings of Rasheed [52] in a study among female students in Saudi Arabia, in which women preferred dieting to exercise to lose weight. According to that study women had a poor attitude towards exercise. In a study in Ghana, which assessed the healthy life behaviours among a sample population, men were found to have a higher physical activity level than women [53]. Several other studies have also reported women having low physical activity levels compared with men, indicating the low preference of women for exercise [7, 42, 53].

The inability of participants in this study to self-perceive themselves as overweight/obese can hinder the effectiveness of weight control programmes because they will have less incentives to control weight [45]. Social determinants of weight perception are of clinical and public health significance [45, 54] and further research is recommended to explore the macro and micro social processes involved. This will help curb the rising prevalence of overweight/obesity in Ghana.

It is critical to note that this study is subject to some limitations. This study did not consider socio-economic status of the participants which have been shown to influence weight perception. Some questions on socio-economic status were asked but the responses were either missing or ambiguous. Secondly, our study did not consider body size and body image perception which are important factors of weight perception and weight loss attitudes. Thirdly, this study was carried out on a small sample in an urban area and as a result the findings cannot be generalized to the entire Ghanaian population. However, it can serve as a reference point for further studies in the country. Finally, the cross-sectional nature of this study cannot investigate the direction of causality.

## Conclusion

A high prevalence of *central obesity* and distorted self-perceived weight status was observed. Weight loss attitudes were not associated to distorted self-perceived weight status. Distorted self-perceived weight status was common among *overweight/obese* participants, younger participants and participants who lacked knowledge on the health effects of overweight and obesity. Weight perception should be considered in designing weight loss intervention in the future. In addition, people should be educated on the health effects of overweight and obesity. These are important strategies that should be considered in addressing the rising prevalence of overweight and obesity in Ghana.
